# Extracellular Vesicles-Mediated Bio-Orthogonal Catalysis in Growing Tumors

**DOI:** 10.3390/cells13080691

**Published:** 2024-04-16

**Authors:** Maria Sancho-Albero, Victor Sebastian, Ana M. Perez-Lopez, Pilar Martin-Duque, Asier Unciti-Broceta, Jesus Santamaria

**Affiliations:** 1 Instituto de Investigación Sanitaria de Aragón (IIS Aragón), Avda. San Juan Bosco, 13, 50009 Zaragoza, Spain; victorse@unizar.es (V.S.); jesus.santamaria@unizar.es (J.S.); 2 Instituto de Nanociencia y Materiales de Aragón (INMA), CSIC-Universidad de Zaragoza, Campus Rio Ebro, Edificio I+D, C/Poeta Mariano Esquillor, s/n, 50018 Zaragoza, Spain; 3 Networking Research Center in Biomaterials, Bioengineering and Nanomedicine (CIBERBBN), Instituto de Salud Carlos III, 28029 Madrid, Spain; 4 Department of Chemical and Enviromental Engineering, University of Zaragoza, Campus Rio Ebro, C/María de Luna, 3, 50018 Zaragoza, Spain; 5 Edinburgh Cancer Research, CRUK Scotland Centre, Institute of Genetics & Cancer, University of Edinburgh, Edinburgh EH4 2XR, UK; anamperezlopez@gmail.com (A.M.P.-L.); asier.ub@ed.ac.uk (A.U.-B.); 6 Instituto de Salud Carlos III, 28222 Madrid, Spain

**Keywords:** extracellular vesicles, bio-orthogonal catalysis, cancer, palladium nanosheets

## Abstract

Several studies have reported the successful use of bio-orthogonal catalyst nanoparticles (NPs) for cancer therapy. However, the delivery of the catalysts to the target tissues in vivo remains an unsolved challenge. The combination of catalytic NPs with extracellular vesicles (EVs) has been proposed as a promising approach to improve the delivery of therapeutic nanomaterials to the desired organs. In this study, we have developed a nanoscale bio-hybrid vector using a CO-mediated reduction at low temperature to generate ultrathin catalytic Pd nanosheets (PdNSs) as catalysts directly inside cancer-derived EVs. We have also compared their biodistribution with that of PEGylated PdNSs delivered by the EPR effect. Our results indicate that the accumulation of PdNSs in the tumour tissue was significantly higher when they were administered within the EVs compared to the PEGylated PdNSs. Conversely, the amount of Pd found in non-target organs (i.e., liver) was lowered. Once the Pd-based catalytic EVs were accumulated in the tumours, they enabled the activation of a paclitaxel prodrug demonstrating their ability to carry out bio-orthogonal uncaging chemistries in vivo for cancer therapy.

## 1. Introduction

Cancer is a multifaceted global health issue that continues to demand new solutions and represents one of the greatest challenges in the biomedical field all around the world [1,2]. By 2040, the cancer burden is expected to reach 28.4 million cases worldwide, which represents a 47% increased incidence in 20 years [3]. In spite of the wide array of conventional and emerging therapies, the treatment of certain cancers is not curative and is only able to delay disease progression or palliate symptoms. This lack of treatment efficiency often relies on the significantly low accumulation of the antitumoral molecules or therapies in the cancerous tissues, which limit their therapeutic window. Therefore, there is significant interest in improving current anticancer therapies (particularly chemotherapy) to selectively and effectively kill cancer cells without causing damage to healthy tissues.

Nanomedicine was heralded as one of the potential solutions to this problem, and indeed, a large variety of nanovectors have been developed with exciting therapeutic properties [4,5,6]. Particularly promising are metal-based nanoparticles (NPs), that present exciting advantages, including narrow size and shape distribution, long activity periods, dense surface functionalization and capability for optical or heat-based therapeutic strategies [7,8]. More interestingly, metallic nanomaterials (gold, platinum, palladium or silver NPs) are emerging as alternative agents for cancer therapy because of their catalytic properties under physiological conditions, which are able to induce deep changes in the tumour microenvironment (TME) and lead to cancer cell death by various strategies [9,10].

Among the catalytic approaches for addressing cancer, the emerging field of bio-orthogonal uncaging reactions seems particularly promising [11,12,13]. Bio-orthogonal catalysis aims to enable bio-independent chemical reactions, to obtain information about specific biochemical processes or to produce bioactive molecules and drugs capable of modulating physiological and pathological processes [14,15]. A number of metal-based NPs have been reported as excellent catalysts to activate intracellular bio-orthogonal reactions. Among these, Pd-based catalysts have played a central role in view of the wide variety of processes that can be addressed with them, including dealkylations, [16,17,18,19,20] cross-linking reactions, [21,22,23,24] ring formation, [25] cleavage reactions, [26] etc., as well as uncaging bioactive products with different functional groups [27,28,29]. In this context, the synthesis of Pd catalysts in the form of palladium nanosheets (PdNSs) adds unique physical and chemical properties: surface plasmon resonance (SPR) effect (that provides light absorption in the near infrared region (NIR) and therefore, the possibility of doing optical hyperthermia therapy) and high stability in solution. Clearly Pd-catalyzed chemistry has the potential to generate cytotoxic species and other functional molecules in situ, reducing possible side effects in healthy tissues. Following this approach, we have demonstrated the use of palladium (Pd) nanoparticles to uncage clinically approved therapeutic molecules for anticancer applications [17,19,20,30,31,32].

However, although several proofs of concept have demonstrated promising results regarding the use of bio-orthogonal catalysis in vitro and in vivo, [33,34,35] there are still important limitations regarding the delivery of the catalyst only to the target tissues and cells. This is especially important in cancer where, even in the case of active targeting (using specific antibodies or sugars), the proportion of nanoparticles reaching the tumour is low (around 1% of the administered dose) [36]. Thus, despite their interesting properties as anticancer tools, their translation to clinic has largely failed due to the lack of specific delivery to the tumors [37,38]. It seems clear that more specific approaches for selective delivery to cancer sites are sorely needed.

Among the most promising strategies, extracellular vesicles (EVs), are being investigated as a possible solution to the targeting challenge. EVs are key elements for communication between cells [39]. Of particular interest are exosomes, nanovesicles (50–150 nm in diameter) of an endocytic nature secreted by almost all cell types [40]. These vesicles are formed by (1) a cytosolic inner compartment where proteins, nucleic acids and biomolecules are located and (2) a protein-phospholipidic double membrane. Their high potential in targeting stems from properties associated with their differential composition, giving a preferential tropism towards the secreting cell lines [41,42]. In view of this, cancer-derived EVs present attractive properties as vectors for the efficient delivery of a variety of therapeutic nanomaterials to treat cancer [43].

In previous work we have prepared Pd and Pt-loaded EVs with the aim of creating hybrid vectors with catalytic and improved targeting properties [42,44,45]. To preserve the targeting properties of the membrane we developed a mild reduction procedure using CO as the reducing agent to fabricate nanoparticles from noble metal precursors previously infiltrated on the EVs. In the case of Pd this gave rise to exosomes with a high load of Pd nanosheets (Pd-EVs). Encouraged by the promising results obtained with these catalytic EVs in cell culture models, here for the first time we explore the possibility of using cancer derived Pd-EVs as in vivo therapeutic vectors to carry out prodrug uncaging reactions in animal models.

## 2. Materials and Methods

### 2.1. Synthesis of PEG-Coated Pd Nanosheets (PEG-PdNSs)

All the chemicals were provided by Sigma Aldrich (St. Louis, MO, USA). The production of ultrathin Pd nanosheets (PdNSs) was based on previously reported protocols [46,47,48] but avoiding the use of toxic quaternary ammonium salts. The palladium growth solution was prepared by mixing 11.5 mg of Na_2_PdCl_4_, 33 mg of poly(vinyl pyrrolidone) (MW = 55,000) and 120 mg of KBr in Milli-Q water (400 μL). The resulting homogeneous red solution was mixed with 4 mL of dimethylformamide (DMF). The Pd nanosheet precursor solution was homogenized in an ultrasound bath and introduced into a high-pressure stainless-steel Teflon-lined reactor. A CO gas atmosphere (6 bar) was set inside the reactor to reduce the Pd precursor and promote the anisotropic growth of Pd into very thin nanosheets of around 1.5 nm [49]. The reactor was placed in a heated water bath (80 °C for 40 min) and afterwards it was cooled down. A dark blue colloid was obtained after the CO treatment. The resulting PdNSs were collected by centrifugation (7000 rpm, 10 min) by mixing the dark blue colloid and acetone in a volume ratio of 1 to 3. The resulting PdNSs were redispersed in Milli-Q water to be functionalized with polyethylene glycol (PEG) to obtain PEG-PdNSs using an excess of monofunctional poly(ethylene glycol)-ether thiol (PEG 800 mW), taking advantage of the strong chemical bond between Pd and S. With this aim, a solution of PdNSs was put in contact with a dilution of PEG (1:1 *w*/*w*) for a duration of 30 min under magnetic stirring conditions. Then, any excess of unbound PEG was removed by dialysis against distilled water. The resulting PdNSs and PEG-PdNSs were finally characterized by transmission electron microscopy (TEM), ultraviolet-visible (UV-VIS) spectroscopy, Fourier-transform infrared spectroscopy (FTIR) and microwave plasma atomic emission spectroscopy (MP-AES) as described in the next section. Also, the electrokinetic potential was estimated by ζ potential measurements at pH = 5.5 in Milli-Q water (Brookhaven 90 plus and PALS Zeta Potential Analyzer 2.5).

### 2.2. Fabrication and Characterization of Pd-Loaded EVs (Pd-EVs)

A549 cells (kindly provided by Dr S. Wilkinson; ATCC, CCL-118) were cultured in Dulbecco’s modified Eagle’s medium (DMEM) supplemented with 10% fetal bovine serum (FBS), 1% penicillin/streptomycin and 1% amphotericin, under normoxic conditions. Cells were checked for mycoplasma before use. Endogenous EVs of the FBS were previously removed by ultracentrifugation (100,000, 8 h and 4 °C).

EVs were collected and purified by successive ultracentrifugation cycles from the supernatants of A549 cells at confluency [42]. Briefly, a first cycle at 2000× *g* and 4 °C for 20 min was carried out to discard cell fragments and debris. Then, a second centrifugation step was carried out at 10,000× *g* and 4 °C for 30 min to sediment and discard organelles and microvesicles. Finally, to isolate the EV fraction, samples were ultracentrifuged at 100,000× *g* and 4 °C for 2 h. The obtained EV pellet was suspended in phosphate-buffered saline (PBS) and ultracentrifuged again (100,000× *g*, 2 h and 4 °C) in order to eliminate the proteins superficially bounded to the vesicles. Isolated EVs were suspended in PBS and characterized by a broad battery of techniques. A Pierce BCA protein assay was carried out following manufacturer instructions in order to estimate the protein content and to quantify the EVs. The morphology, shape and size of the isolated EVs were characterized by TEM. A total of 5 μL of EV samples was deposited onto a copper TEM grid before being contrasted with phosphotungstuc acid (3%). Samples were visualized by TEM (T-20 FEI Technai transmission electron microscopy) operated at 200 kV with an LaB6 electron source fitted with a SuperTwin^®^ objective lens allowing a point-to-point resolution of 2.4 Å (T20-FEI).

To load EVs with PdNSs, the protocol designed by our group was followed [42,44]. In brief, the isolated EVs were dispersed in a PBS solution and incubated with K_2_PdCl_4_ (0.06 mM) at room temperature for 12 h to favor the internalization and diffusion of Pd^2+^ species to the internal cavity of the EVs. Then, the mixture was ultracentrifuged (100,000× *g*, 2 h and 4 °C) to discard the non-internalized Pd ions and avoid the formation of non-internalized metal NPs during the reduction step. Pd^2+^-EVs were suspended in PBS and treated for 40 min at 6 bar under CO atmosphere in a Teflon-lined autoclave under gentle stirring and 40 °C to reduce the Pd^2+^ species into Pd^0^. After the treatment, CO was replaced by air. 

Pd-EVs were characterized in terms of morphology, shape and size by BCA, TEM and Zeta potential as previously described. An Analytical Titan (FEI company) high resolution transmission electron microscope with a spherical aberration corrector was used for high-angle annular dark-field scanning transmission electron microscopy (HAADF-STEM) imaging at 300 kV. Energy dispersive X-ray spectroscopy (EDS) analysis was performed to determine the presence of the PdNSs inside the EVs. NTA analysis was performed to evaluate the hydrodynamic diameter and the number of particles per mL of the control EVs and the Pd-EVs. Furthermore, the amount of Pd inside the vesicles was measured using MP-AES (4100 MP-AES, Agilent Technologies, Santa Clara, CA, USA) and normalized by the total protein amount of Pd-EVs. To do that, samples were digested with 10% aqua regia (HNO_3_ + 3HCl) in 1.5 mL of dH2O for a duration of 2 h at room temperature. Calibrations were carried out employing a Pt standard in 10% aqua regia ranging from 0 to 10 ppm. The characterization of the absorbance properties of Pd-EVs was determined by UV-VIS spectroscopy (Jasco V670) to identify the characteristic plasmon at the NIR range of PdNSs. The synthesis of these materials has been performed by the ICTS “NANBIOSIS”, more specifically by the Synthesis of Nanoparticles Unit (UNIT 9) of the CIBER in Bioengineering, Biomaterials and Nanomedicine (CIBER.BBN).

### 2.3. Synthesis of Pro-PTX

**Scheme 1:**
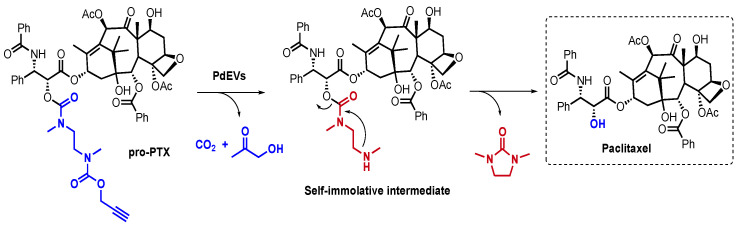
Pd-mediated *O*-depropargylation of pro-PTX followed by a spontaneous intramolecular cyclization to generate paclitaxel and the non-toxic by-product 1,3-dimethyl-2-imidazolidinone.

The prodrug of paclitaxel (pro-PTX) was prepared following established protocols [30], yielding a compound with spectral characteristics consistent with literature values, indicating >95% purity by both high-performance liquid chromatography (HPLC) and proton nuclear magnetic resonance (1H-NMR) analyses. In brief, 2′-(4-Nitrophenoxycarbonyl)paclitaxel (269 mg, 1 eq) was dissolved in dry DMF (15 mL) under a N2 atmosphere and cooled to 0 °C. Tert-butyl methyl [2-(methylamino)ethyl]carbamate (134 mg, 3 eq) and *N*,*N*-Diisopropylethylamine (DIPEA) (230 μL, 5 eq) were dissolved in dry DMF (10 mL) and added dropwise to the solution. The mixture was allowed to warm to room temperature and stirred overnight. After the removal of the solvent by rotary evaporation, the crude product was purified by semipreparative TLC chromatography. With Pd catalysts, the pro-PTX produces paclitaxel following a depropargylation reaction (Scheme 1):

### 2.4. Animal and Tumor Model Optimization

All of the procedures were carried out following the rules and guidelines governing the use and care of laboratory animals and under the Project License PI 45/20 approved by the Ethic Committee for Animal Experiments from the University of Zaragoza. Mice were fed ad libitum and their care and maintenance under specific pathogen-free conditions were carried out accordingly with the Spanish Policy for Animal Protection RD53/2013, which meets the European directives and guidelines Union Directive (EEC Council Directive 2010/63/UE).

For the experiments, 6–8-week-old female BALB/c nu/nu mice (Envigo) were used. The animals were maintained under quarantine for 7 days as soon as they arrived at the animal facilities and before starting the experiments. For the tumour transplant, the animals received an injection of 7.5 × 10^6^ A549 cells in PBS mixed with Matrigel (cells:Matrigel 1:1) in 200 μL as the final volume.

### 2.5. Biodistribution of Pd-EVs and PEG-PdNSs and Tolerability of the Pro-PTX

**Figure 1:**
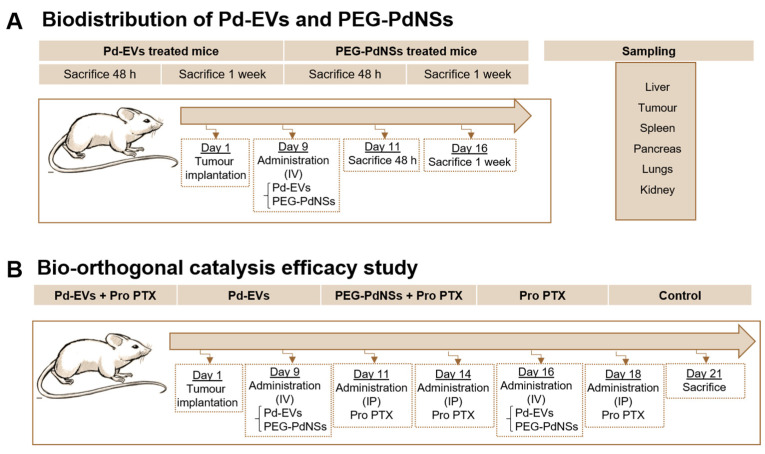
Experimental procedures followed in the in vivo experiments. (**A**) Overall design of the study regarding the biodistribution of Pd-EVs and PEG-PdNSs. (**B**) Overall design of the study to test the bio-orthogonal catalysis therapy in vivo.

In the biodistribution studies, after tumour implantation mice were randomly divided into four groups. Each group represented a different time-point of sacrifice for both vectors administered: (1) Pd-EVs and (2) PEG-PdNSs. A total of 100 μg of Pd-EVs (expressed in terms of total protein amount obtained by BCA and containing 0.15 μg of Pd/μg of EV) were administered in the tail vein and 48 h and 1 week after their administration, mice were sacrificed. The PEG-PdNS-treated mice received the same amount of Pd catalyst (15 μg of Pd/mice) as the Pd-EVs-treated animals. This group was used as the control, comparing the biodistribution of the PdNSs when they were PEGylated with the EV-encapsulated ones. The experimental plan containing animal groups, time points and sampling is included in Figure 1A.

Tumour, kidneys, liver, lungs, spleen and pancreas were collected from each animal for quantifying the catalyst accumulation (in terms of Pd amount) by ICP-MS (inductively coupled plasma mass spectrometry). To quantify the amount of Pd, each organ was digested with 1 or 3 mL (depending on organ volume) of aqua regia for a duration of 5 days at room temperature. Then, samples were filtered with a 0.2 syringe filter and diluted in miliQ H_2_O. Finally, the total amount of the metal present in the tissues was determined by ICP-MS (Perkin Elmer Elan DRC-e) in the Chemical Analysis Service from the University of Zaragoza.

The membrane of the EVs was fluorescently labelled with the dye Claret following a previously optimized protocol [50]. An immunofluorescence analysis was carried out to determine the presence of fluorescence signal in the tumour by confocal microscopy (Zeiss LSM 800). With this aim, samples were frozen with isopentane cooled by liquid nitrogen and cryosectioned at five micrometers. Slices were assembled in a slide with FluoromentG and DAPI (ex/em 364/454) for visualization. In the confocal microscope analysis, a multialignment of 10 × 10 images with the 20× objective were performed to visualize the whole tissue. Pd-EVs were visualized due to their fluorescence from the Claret probe (ex/em 655/675).

To determine the tolerability of the prodrug of paclitaxel (PTX), 10 mg/kg and 20 mg/kg of the molecule were intraperitoneally administered twice every three days. One week after the first administration, animals were sacrificed. Immediately, blood was extracted and the hepatic profile of control and prodrug-treated animals was assessed using a VetScan VS2. In particular, the following biomarkers were analyzed: ALP (alkaline phosphatase), ALT (alanine Aminotransferase), GGT (gamma glutamyl transferase), BA (bile acids), ALB (albumin), BUN (blood urea nitrogen and CHOL (total cholesterol).

### 2.6. Therapy Effiacy Based on Bio-Orthogonal Catalysis

To explore the efficacy of the bio-orthogonal catalysis mediated by the Pd-EVs as an antitumoral tool, the animals were divided in five groups: group (1) non-treated animals; group (2) control mice treated with the prodrug; group (3) control mice treated with the Pd-EVs; group (4) mice treated with PEG-PdNSs and the prodrug; and group (5) mice treated with the Pd-EVs and the prodrug.

Nine days after tumour implantation, the Pd-EVs and the PEG-PdNSs were intravenously administered as previously described to mice from groups 4 and 5. Two and five days after the catalyst administration, the prodrug was intraperitoneally administered (20 mg/kg). After one week, a second administration of catalysis was applied and an additional administration of prodrug was also carried out two days after. Tumour size was periodically evaluated using a caliper. Mice weight was also monitored. The experimental plan containing animal groups and the time points is included in Figure 1B.

### 2.7. Statistical Analysis

The results are expressed as ±SD. A statistical analysis of the data and the significant differences among the means was performed. Data followed a normal distribution and their statistical analysis was carried out by one way and two-way analysis of variance (ANOVA) for multiple comparisons using GraphPad Prism 8.0 Software. Statistically significant differences were expressed as follows: * *p* < 0.05; ** *p* < 0.01; *** *p* < 0.0001; and y **** *p* < 0.00001.

## 3. Results and Discussion

### 3.1. Characterization of Pd-Loaded EVs (Pd-EVs) and PEG-PdNSs

**Figure 2:**
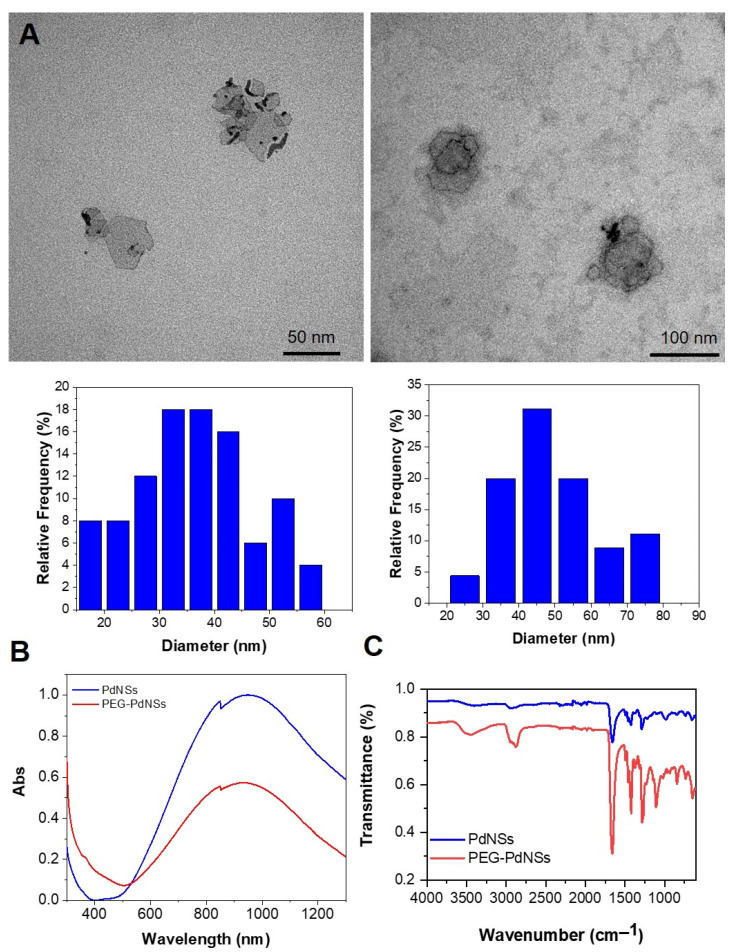
Characterization of PdNSs and PEG-PdNSs. (**A**) TEM image of PdNSs (**left**) and PEG-PdNSs (**right**) produced with a CO pressure of 6 bar after 40 min at 25 °C. PEG functionalization is clearly visible around the nanosheets by a negative staining to contrast the organic shell. Size distribution diagram of NPs obtained from TEM images (bottom). (**B**) UV–VIS NIR spectrum of PdNSs dispersed in water. (**C**) FTIR spectra of naked PdNSs and PEG-coated PdNSs.

Figure 2A shows images of the as prepared PdNSs (left) and the PEGylated PdNSs (right). The obtained structures exhibited a sheet morphology with a mean thickness of around 1.5 nm, in concordance with previously published results [46,49]. When NPs were functionalized with PEG, the organic shell around them could be clearly observed after staining. TEM images at a lower magnification of PdNSs and PEG-PdNSs are shown in Figure S1. EDS confirmed the presence of Pd atoms in the ultrathin nanosheets (Figure S2). The mean diameter of PdNSs obtained from the TEM images was 36.4 ± 10.8 nm, whereas the mean size of PEGylated PdNSs (determined by negative staining the TEM sample) was 46.4 ± 21.6 nm. The ultrathin morphology of the PdNSs and the PEG-PdNSs gave rise to specific optical properties causing absorbance in the NIR region as measured (Figure 2B). From Figure 2B we can also conclude that when the PdNSs were PEGylated their absorption properties were modified. Finally, the presence of the PEG around the nanosheets was characterized by FTIR analysis (Figure 2C). The FTIR spectrum of the PdNSs exhibited the characteristic peaks coming from the PVP (employed as a stabilizer during the PdNSs synthesis). In particular, characteristic bands at 1292, 1441, 1669 and 2930 cm^−1^ were observed [51]. When the PdNSs were PEGylated, the PEG characteristic peaks at 3450 cm^−1^ (OH stretching), 2868 cm^−1^ (SH stretching), 1458 cm^−1^ (CH bending vibrations from CH2 groups) and 1248 cm^−1^ (C-O stretching vibration) were also evidenced in the spectra [52]. Finally, the surface charge of the nanostructures was also determined. Zeta potential measurements of PdNSs and PEG-PdNSs yielded values of −8.13± 0.35 mV and −12.79 ± 0.67 mV, respectively. 

**Figure 3:**
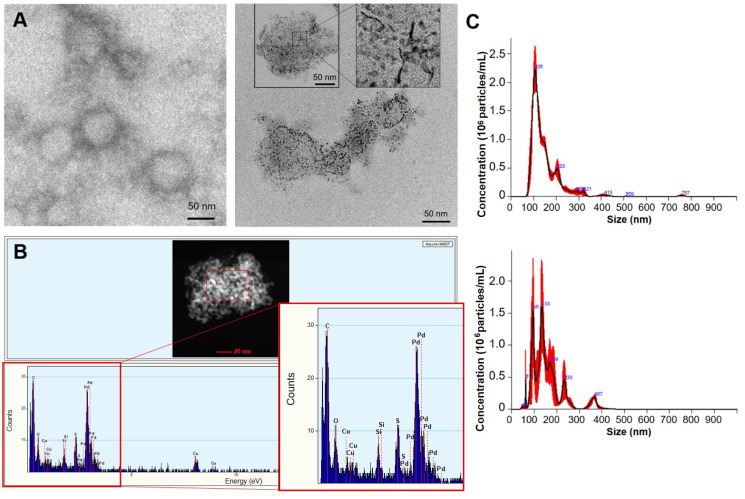
Characterization of EVs and Pd-EVs. (**A**) Representative TEM images of EVs negatively stained with phosphotungstic acid. Inset with a PdNS EV at high magnification. (**B**) HAADF-STEM images of Pd-EVs and EDS spectrum of the red rectangle area. (**C**) Particle size analysis of non-loaded and Pd-loaded EVs obtained by NTA. It can be seen that the agglomeration (higher size peaks in NTA) increases somewhat for the Pd-EVs; however, both empty and loaded EVs exhibited comparable mean hydrodynamic diameter.

EVs were characterized before and after CO infusion by several physicochemical techniques, including TEM, HRSTEM-HAADF and NTA. Round-shaped vesicles with an average diameter of 100–140 nm were observed in the empty and in the Pd-loaded samples. The dark contrast observed in TEM images stems from the presence of Pd nanostructures with higher electron-dense atoms than that of the EVs. The Pd nanostructures have a mean size of approximately 10 nm and a thickness of 1.4 nm (observed in those nanosheets that are tilted in the image), which is in agreement with previous results [48]. PdNSs were only observed in association with the EVs, inferring that the loading process and purification process had been carried out efficiently (Figure 3A). The loaded EVs containing such nanostructures did not exhibit morphological or size alterations compared to the empty ones. HAADF-STEM and EDS analyses also confirmed the presence of tiny PdNSs inside the EVs (Figure 3B). Finally, NTA analysis revealed that neither the diameter (approx. 130 nm) nor the concentration (approx. 2 × 10^6^) of the EVs were strongly affected by the treatment with CO in the presence of the Pd precursor and the subsequent generation of the PdNSs (Figure 3C).

### 3.2. Biodistribution of Pd-EVs and PEG-PdNSs and Tolerability of the Prodrug

**Figure 4:**
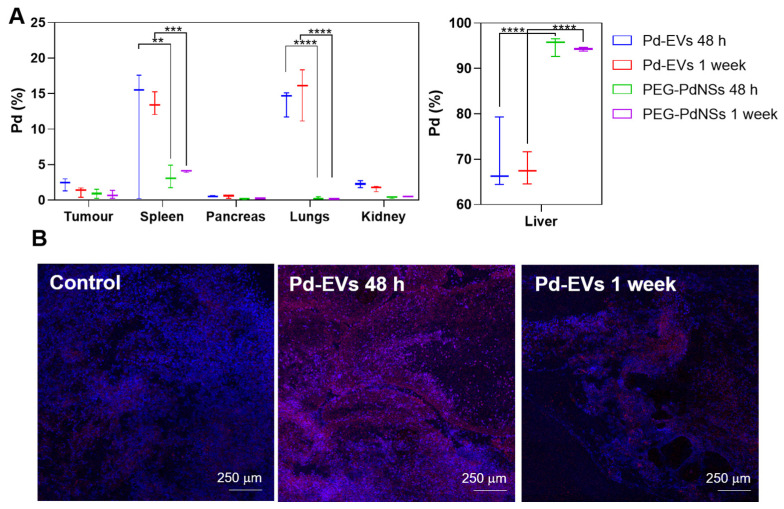
Biodistribution of Pd-EVs and PEG-PdNSs in A549-tumor bearing mice. (**A**) ICP-MS analysis of total Pd found in organs extracted from mice after 48 h and 1 week Pd-EVs and PEG-PdNSs intravenous administration. Pd amount is expressed in terms of% (based on total Pd detected). (**B**) Ex vivo fluorescence multialignament images of xenograft tumors 48 h and 1 week after intravenous administration to the Pd-EV mice and control (non-treated) mice. The cell nucleus was marked with DAPI (blue) and the fluorescently labelled Pd-EVs are observed as red color aggregates. ** *p* < 0.01; *** *p* < 0.0001; and y **** *p* < 0.00001.

The in vivo biodistribution of Pd-EVs intravenously administered was evaluated in tumour-bearing mice. PEG-PdNSs were administered as a reference for comparison, since their accumulation in the tumour would be driven by the EPR effect. The presence of both PEG-functionalized and EV-coated PdNSs in the different organs was quantified by ICP-MS in terms of Pd amount after organ digestion. Figure 4A reveals that liver, lungs and spleen were the main accumulation tissues of both Pd-EVs and PEG-NSs. The statistical analysis of these results was performed organ by organ comparing the amount of Pd accumulated in the organs from mice treated with PEG-PdNSs and Pd-EVs after 48 h and 1 week from their administration. These results revealed a strong increase in Pd-EVs in the spleen and lungs compared to the PEGylated PdNSs. On the contrary, a significantly lower accumulation occurred in the liver when the PdNSs were delivered within the EVs (94.98% and 94.26%. of the Pd was accumulated in the liver 48 h and 1 week after the administration of the PEG-PdNSs, whereas this amount significantly decreased to 70.0% and 67.89% for EVs-PdNSs).

In the tumour, 0.91% of the Pd was found 48 h after the administration of the PEG-PdNSs, broadly in agreement with the expectations for the EPR effect [36]. However, when the same amount of Pd was administered inside EVs, the delivery to the tumour was 2.5 times higher (2.25%). After one week, the Pd amount measured in the tumor was still significantly higher for Pd-EVs compared to PEG-PdNSs: 1.18% and 0.76%, respectively. Taken together, these results clearly point to a rather different biodistribution depending on the mode of delivery used. Pd-EVs evidenced a very significant improvement in the accumulation in the tumor compared to the enhanced permeability and retention (EPR) effect, together with an increase in the lung and spleen and a statistically significant decrease in Pd-EVs located in the liver compared to the PEGylated nanocarriers.

In addition, Pd-EVs were fluorescently labelled with a Claret probe (ex/em 655/675) and their biodistribution was also assessed ex vivo by confocal fluorescence microscopy (Figure 4B). The immunofluorescence analysis of the tumors corroborated the high concentration of Pd-EVs in the cancer tissue. In agreement with ICP-MS results, the Pd-EV content in tumors was higher 48 h after their administration compared to the one-week time-point.

The decrease in metal nanoparticles in the liver when administered as PdEVs is considered valuable, as this organ has a significant role in the metabolism, detoxification and elimination of substances from the body. This is particularly important in this study in which we aim to use a non-toxic pro-PTX developed by our lab [48]. PTX is one of the most widely used chemotherapy drugs in cancer treatment, including lung cancer.

**Figure 5:**
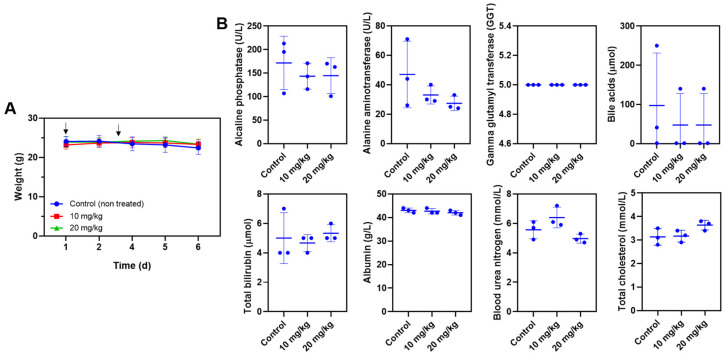
(**A**) Weight evolution of mice and (**B**) plasma levels of alkaline phosphatase (ALP), alanine aminotransferase (ALT), gamma glutamyl transferase (GGT), bile acids (BA), total bilirubin (TBIL), albumin (ALB), blood urea nitrogen (BUN) and total cholesterol (CHOL) in non-treated control mice and in mice treated twice (separated by three days) with the pro-PTX at 10 mg/kg and 20 mg/kg.

To evaluate the tolerability of the pro-PTX in vivo, mice were treated with pro-PTX twice every three days. One week after the second administration, mice were sacrificed and blood was extracted. The corresponding data sets of enzyme activities (ALP, ALT) GGT, BA, TB ALB, BUN and lipids (CHOL) were analyzed. Elevations in ALT, AST, ALP and total bilirubin denote hepatocellular disease. The evaluated parameters did not demonstrate significantly different levels between the different groups. In fact, 10 mg/kg and 20 mg/kg of pro-PTX treated mice exhibited similar levels of ALP, ALT GGT, BA, ALB, BUN and CHOL compared to the non-treated mice (Figure 5). These parameters were within the reference intervals established for clinical chemistry in mice [53,54]. Therefore we can conclude that the pro-drug of PTX did not cause significant hepatocellular toxicity in mice.

### 3.3. Bio-Orthogonal Catalysis Efficacy In Vivo

**Figure 6:**
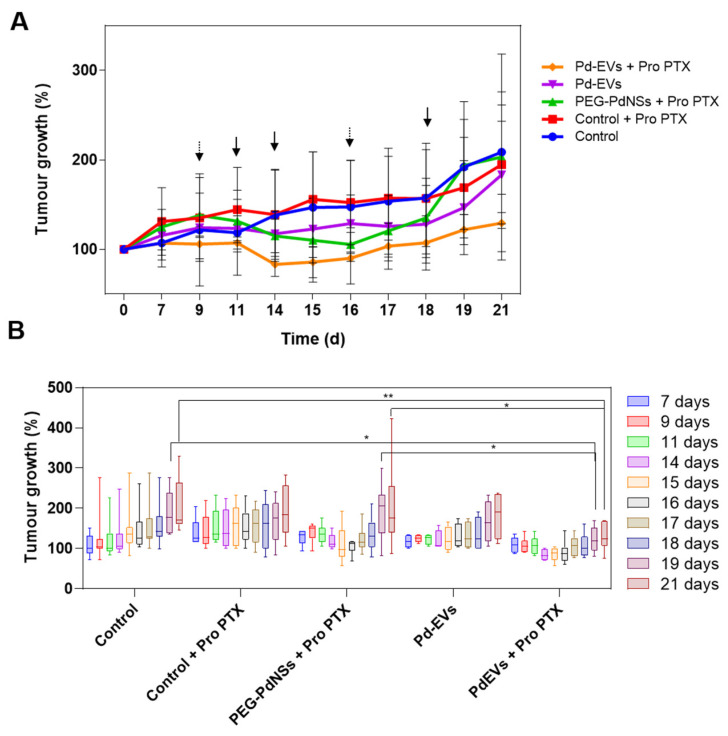
Bio-orthogonal therapy on xenograft-bearing mice. (**A**) Tumour size evolution of the different treated and control groups. The time-points of the two IV administrations of Pd-EVs or PEG-PdNSs (dot point arrows) and the corresponding pro-PTX intraperitoneal administration (continuous arrows). (**B**) Statistical analysis of tumour size for each animal group. * *p* < 0.05, ** *p* < 0.01.

Once the tolerability of the prodrug was demonstrated, tumor-bearing mice were treated with the prodrug and with the Pd catalyst, which would convert it into paclitaxel. Tumor growth was monitored until they reached the ethically allowed size by the local committee. As Figure 6A,B indicates, tumor development for animals of the three control groups (non-treated mice, pro-PTX treated mice and Pd-EV treated mice) increased progressively over time. In the case of PEG-PdNS-treated mice in combination with the pro-PTX, their tumor size seemed to be stopped after the first administration of catalyst and prodrug. However, at day 16, tumor sizes from this group started to grow rapidly, showing similar sizes to control groups at the endpoint of the study. In contrast, the tumors of animals treated with Pd-EVs and pro-PTX stopped growing and even decreased in size after the first administration. Then tumor growth continued and after the second administration of the prodrug in combination with the catalyst, the growth rate was significantly slower compared with the other mice groups.

The differences found in antitumoral efficiency between Pd-EVs and PEG-PdNSs (same catalyst, same prodrug) can only be interpreted in terms of the delivery achieved in the case of Pd-EVs, with 2.5 times higher delivery. In the case of PEG-PdNSs the amount of catalyst accumulated in the tumor is not enough to produce paclitaxel in sufficient amounts. These findings indicate that EVs not only deliver PdNSs to tumor tissue more efficiently, but also that the PdNSs inside the vesicles preserve their catalytic properties for a time sufficient to enable the bio-orthogonal uncaging reactions in vivo to convert the pro-PTX to paclitaxel in sufficient quantities to produce a substantial slowing of tumor growth.

The results of this work contribute to the validation of the “Trojan Horse” concept as an emerging strategy for enhancing tumor-targeting efficiency [55,56]. Among “Trojan Horse” strategies, EVs clearly have a strong potential as tools for solving the targeting challenge [57,58]. For instance, Sedeghi et al., reported the use of thermostable self-assembling porous exoshells to encapsulate and deliver an iron-containing reaction center for the treatment of breast cancer [59]. In other recent work, noble metal NPs able to carry out bio-orthogonal depropargylations with high efficiency in biological media were encapsulated in mesoporous silica nanorods and in a biodegradable PLGA matrix. These complexes were able to successfully mediate the intracellular uncaging of the clinically approved drug PTX [12].

Five years ago, we reported the first catalytic EVs using the same procedure employed in this work, i.e., a CO atmosphere to induce a mild reduction of infiltrated noble metal precursors, a procedure that is apparently able to preserve membrane integrity [42,50]. This CO reduction strategy therefore represents a very important advance compared to the so-called membrane disruption methods (sonication, freeze-thaw cycles, permeation with saponin or electroporation), that are able to load drugs and very small nanoparticles, but produced serious damage to EV membranes when particles around 40 nm had to be internalized [60]. It also seems superior to the so-called indirect or natural loading strategies that use the natural biogenesis pathway of cells but do not allow for the control of EV loading, giving rise to empty and single multiple-load EVs randomly.

In fact, in the in vivo biodistribution experiments herein reported, although the majority of the palladium still ended in the liver with both Pd-EVs ad PEG-PdNSs, the accumulation of the Pd-EVs in this secondary tissue was statistically significantly much lower compared to the PEGylated PdNSs. In addition, the Pd accumulation in tumor tissue was higher compared with the PEGylated nanosheets. Moreover, we demonstrate how these Pd nanodevices encapsulated in the EVs can perform uncaging chemistries inside cells in vivo, leading to the in situ activation of the anticancer drug PTX and the consequent significant decrease in tumour growth. Taken together, this proof of concept work shed light on the therapeutic potential of catalytic NPs delivered in EVs to carry out bio-orthogonal uncaging chemistries in vivo.

However, even though the amount of catalyst reaching the tumor increased 2.5 times, the biodistribution achieved with Pd-EVs is far from perfect, and is certainly not able to reproduce the selectivity found in in vitro studies [58,60]. In vivo, most of the catalyst ended up in the liver, even for PdEVs, which means that there is ample room for improvement of the delivery efficiency. One of the factors that may be at work here is the morphology of the catalysts used. In the case of Pd nanoslabs, these are relatively large and clustered around the EV membrane, as shown in Figure 3A and in previous work [42]. In contrast, ultrasmall Pt nanoparticles prepared using the same procedure [45], produced a much lower accumulation in the liver after 24 h. This means that the total load, size and shape of the nanoparticles loaded onto EVs may be an important factor to optimize regarding the targeting capacity and the delivery efficiency of EVs.

In summary, the results of the study show that the combination of catalytic PdNSs with EVs significantly hinders tumor growth, improving markedly the results in vivo over those obtained with pegylated nanoparticles (EPR effect). This highlights the potential of this hybrid vehicle as a therapeutic tool in cancer scenarios, thanks to the increased delivery efficiency afforded by the EV membranes. However, the causes that still prevent the translation in vivo of the high selectivity observed in vitro need to be further studied as they may hold the key to the future implementation of Trojan Horse strategies.

## Data Availability

The data that support the plots within this paper and other findings of this study are available from the corresponding author upon reasonable request.

## Supplementary Materials

The following supporting information can be downloaded at: https://www.mdpi.com/article/10.3390/cells13080691/s1, Figure S1: TEM images at lower magnification of PdNSs and PEG-PdNSs; Figure S2: Energy-dispersive X-ray spectroscopy analysis (EDS) of a PdNSs to determine the presence of Pd in the nanostructured.
