# Supplementary material for: Extracellular Vesicles-Mediated Bio-Orthogonal Catalysis in Growing Tumors
Source: Cells. 2024 Apr 16;13(8):691. doi: 10.3390/cells13080691 (PMC11048864; doi:10.3390/cells13080691)
Supplement: Supplementary file 1 [file cells-13-00691-s001.zip › cells-2935537-supplementary.pdf]

SUPPORTING INFORMATION

Extracellular vesicles-mediated bio-orthogonal catalysis in growing tumors

Maria Sancho-Albero[1,2,3,4,*], Victor Sebastian[1,2,3,4] , Ana M. Perez-Lopez[6,7,8], Pilar Martin-Duque[1,3,5*] Asier Unciti-Broceta[6,7], and Jesus Santamaria[1,2,3,4,]

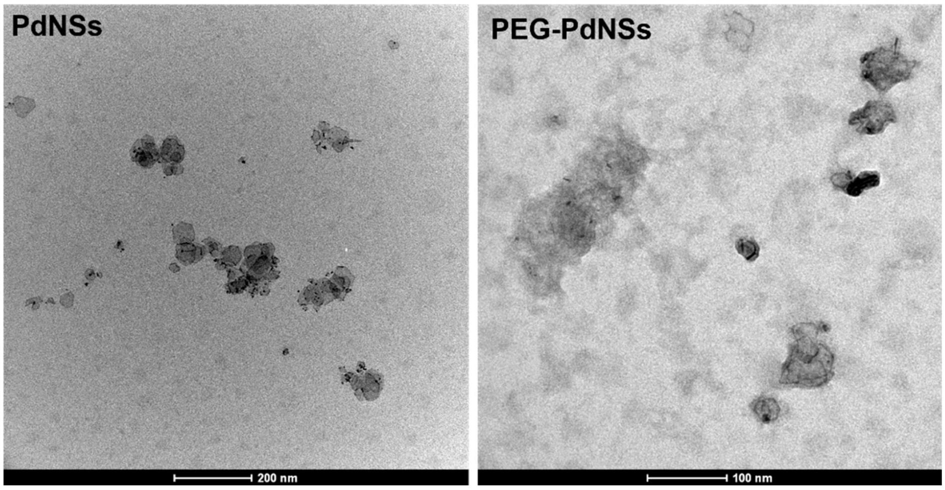

Figure S1. TEM images at lower magnification of PdNSs and PEG-PdNSs.

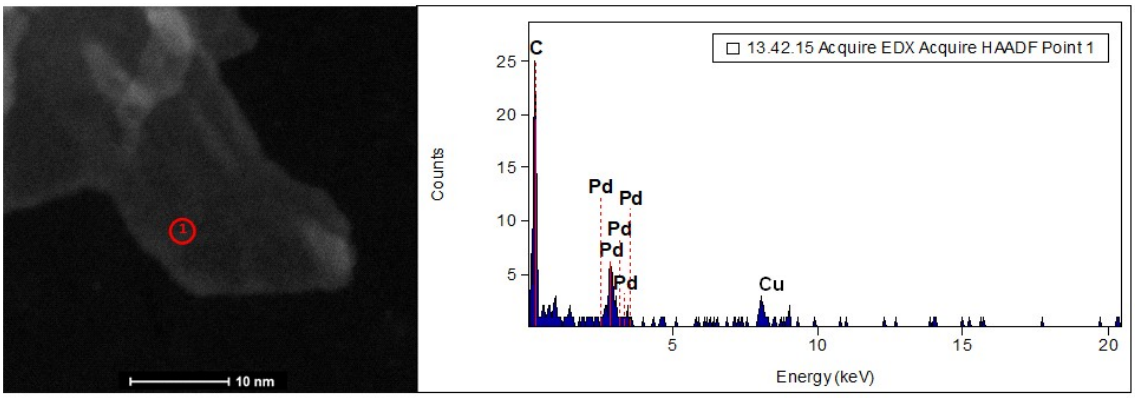

Figure S2. Energy-dispersive X-ray spectroscopy analysis (EDS) of a PdNSs to determine the presence of Pd in the nanostructures. EDS analysis was carried out in the marked area of the STEM-HAADF image.
